# Electro-synthesized Co(OH)_2_@CoSe with Co–OH active sites for overall water splitting electrocatalysis†

**DOI:** 10.1039/c9na00725c

**Published:** 2020-01-06

**Authors:** Yin Wang, Yutong Yang, Xia Wang, Peihe Li, Hongyang Shao, Tianen Li, Haiyang Liu, Qingfu Zheng, Jing Hu, Limei Duan, Changwen Hu, Jinghai Liu

**Affiliations:** Inner Mongolia Key Laboratory of Carbon Nanomaterials, College of Chemistry and Chemical Engineering, Nano Innovation Institute, Inner Mongolia University for Nationalities Tongliao 028000 China jhliu2008@sinano.ac.cn duanlmxie@126.com; Key Laboratory of Cluster Science, Ministry of Education of China, Beijing Key Laboratory of Photoelectronic/Electrophotonic Conversion Materials, School of Chemistry and Chemical Engineering, Beijing Institute of Technology Beijing 100081 China cwhu@bit.edu.cn

## Abstract

Constructing noble metal-free electrocatalytically active sites for the simultaneous hydrogen evolution reaction (HER) and oxygen evolution reaction (OER) in alkaline solution is key to realizing electricity-driven water splitting in practical applications. Here, we rationally designed Co(OH)_2_@CoSe nanorods (NRs) as an excellent bifunctional electrocatalyst by an *in situ* electrochemical transformation strategy, where the Co-based nanorod template was converted into Co(OH)_2_@CoSe at the cathode. The obtained electrode exhibits superior electrocatalytic activity for both the HER (overpotential of 208 mV at 20 mA cm^−2^) and the OER (268 mV at 20 mA cm^−2^) at high current density in a 1 M KOH solution. The theoretical calculations and experimental evidence indicate that the chemical coupling Co–OH active site between Co(OH)_2_ and CoSe regulates the hydrogen adsorption and desorption energy and fast electron transfer capability, which is responsible for the improved HER. Moreover, the Co(OH)_2_@CoSe NRs can be further converted into CoOOH nanosheets which serve as OER active sites. Toward practical electrolytic cell applications, the Co(OH)_2_@CoSe nanorods as both the cathode and anode achieved a current density of 100 mA cm^−2^ at 1.94 V for overall water splitting, better than that of noble metal-based electrocatalysts.

## Abstract 2

An *in situ* electrochemical transformation strategy has been rationally designed to fabricate Co(OH)_2_@CoSe nanorods (NRs) for practical electrolytic overall water splitting.

## Graphical abstract

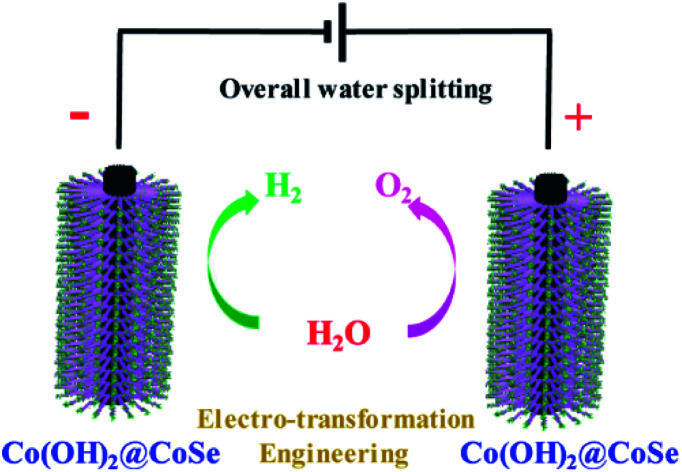

## Introduction

Electricity-driven water splitting is one of the fastest developing energy conversion technologies for renewable energy.^1,2^ The overall water splitting reaction consists of simultaneous electrolysis processes of both the hydrogen evolution reaction (HER) at the cathode and the oxygen evolution reaction (OER) at the anode. Due to the huge energy barriers in the H*/O* adsorption/desorption process and mass transfer resistance under high current during both the HER and OER, efficient electrocatalysts are needed to reduce the overpotential and accelerate the kinetics of the reaction.^3–5^ At present, noble metal-based materials are considered state-of-the-art electrocatalysts for water splitting, *e.g.* Pt-based alloys for the HER^6^ and Ir or Ru-based oxides for the OER.^7^ However, the high cost and scarcity severely hinder their large-scale applications. Recently, some efficient and stable nonprecious metal-based electrocatalysts for the HER in acidic media and the OER in alkaline media have been investigated.^8–12^ However, the problem with these traditional electrocatalysts is that they lack HER activity in strongly basic electrolytes, and good OER catalysts are unstable in strongly acidic solutions.^13–15^ Therefore, it is still a great challenge to develop efficient bifunctional transition-metal active sites to simultaneously realize overall water splitting to H_2_ and O_2_ in the same electrolyte.

Recently, much effort has been focused on transition metal oxides/hydroxides,^16,17^ sulfides,^18,19^ selenide,^20,21^ nitrides,^22^ and phosphides^23–30^ as potential bifunctional electrocatalysts for overall water splitting. In particular, cobalt selenide (CoSe),^31–38^ a typical transition metal chalcogenide, has attracted much attention owing to its excellent HER performance and facile conversion to CoOOH in alkaline electrolytes. But low active surface area and poor conductivity limit the activity of pure CoSe at high current. It is difficult to obtain uniform CoSe nanostructures with a large accessible active surface using conventional methods such as hydro/solvothermal^13,36,39–41^ methods and selenization at high temperature.^15,31,33,35,37^ On the other hand, cobalt hydroxide (Co(OH)_2_) is widely accepted as one of the best-performing OER catalysts.^42–44^ In addition, the HER activity could be promoted by Co(OH)_2_ accelerating the water dissociation process under alkaline conditions.^45,46^ Motivated by these considerations, we assume that the coupling of CoSe with Co(OH)_2_ could generate bifunctional active sites to accomplish overall water splitting in an alkaline electrolyte.

Electrosynthesis is a facile and energy-saving synthetic and preparative technology to obtain electrochemically active materials directly anchored on conducting substrates without extra binders.^47–51^ Sun and co-workers^38^ reported on an electrochemical deposition method to fabricate a CoSe/Ti electrode as a bifunctional electrocatalyst for water splitting. Switzer and co-workers^44^ reported that Co^2+^ and OH^−^ could be electrodeposited onto the cathode to form Co(OH)_2_. In addition, we have developed a modified electrosynthesis method to realize controllable preparation in terms of both the composition and morphology, where a Co-based nanowire precursor was converted into CoO without morphology destruction.^50,51^ These considerations inspire us to exploit an *in situ* electrosynthesis strategy to fabricate one-dimensional (1D) Co(OH)_2_@CoSe nanostructures.

Here, we report a novel *in situ* electrochemical transformation strategy to fabricate Co(OH)_2_@CoSe nanorods (NRs) vertically oriented on conductive carbon cloth (CC). The controllable electrochemical selenization process works at a continuous negative voltage of −0.7 V (*vs.* the saturated calomel electrode, SCE) in a SeO_2_ solution, where Se^2−^ derived from Se^4+^ and OH^−^ from H_2_O combined with Co^2+^ ions from the Co-based nanorod precursor at the cathode. Then, the vertically oriented Co-based nanorods are *in situ* converted into Co(OH)_2_@CoSe NRs. The obtained Co(OH)_2_@CoSe NRs with a large accessible surface area and good charge transport capability exhibit good electrocatalytic activity with small overpotentials of 208 mV for the HER and 268 mV for the OER at a current density of 20 mA cm^−2^ in alkaline solution. Theoretical calculations reveal that chemical coupling Co–OH active site between Co(OH)_2_ and CoSe regulates the H adsorption and desorption energy to promote the HER kinetics, and the CoOOH converted from Co(OH)_2_@CoSe during the OER acts as the real active site for the OER. For an overall water splitting electrolytic cell, the Co(OH)_2_@CoSe NRs need a low voltage of 1.94 V at 100 mA cm^−2^ with catalytic activity and stability much better than those of the (−)Pt/C//RuO_2_(+) cell.

## Results and discussion

**Fig. 1:**
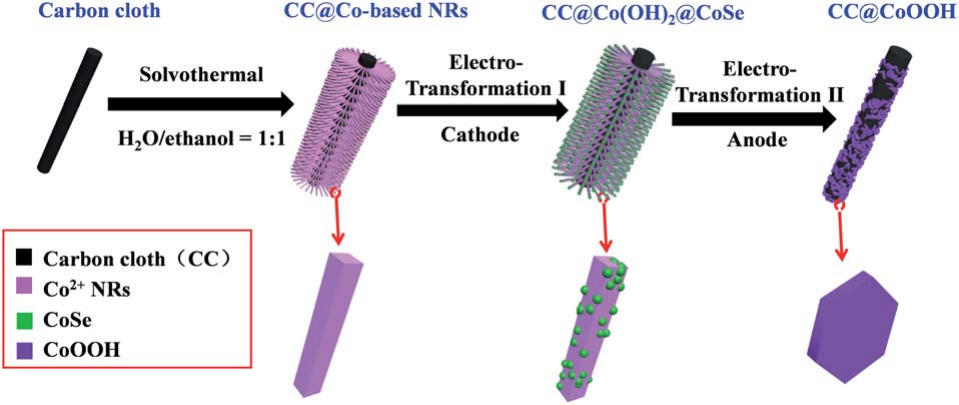
Schematic illustration of the Se redox active *in situ* transformation of CC@Co(OH)_2_@CoSe by the cathode electro-synthesis process and CC@CoOOH by the anode water oxidation process.

*In situ* electrochemical transformation was used to prepare CC@Co(OH)_2_@CoSe nanorods (NRs). As shown in Fig. 1, Co-based NRs were first grown on carbon cloth (CC) as the template through a solvothermal process. The transformation process proceeded at −0.7 V (*vs.* Hg/Hg_2_Cl_2_, SCE) at 60 °C for 40 min in a SeO_2_/KCl solution. Scanning electron microscopy (SEM) images show that the well-defined Co-based nanowires (NWs) and nanorods (NRs) were vertically grown on the surface of carbon cloth after hydrothermal and solvothermal treatment, respectively (Fig. S1 and S2†). Subsequent electrochemical transformation conserves the nanorod morphology with a very rough surface (Fig. S3,†2a and b), while the nanowires were totally destroyed under the same conditions (Fig. S3b†). The size and crystalline structure of individual NRs were investigated by transmission electron microscopy (TEM). The TEM images in Fig. S4† and 2c reveal that the diameter and length are about 300 nm and 4 μm with a mass loading of 1.3 mg cm^−2^ on the carbon cloth.

**Fig. 2:**
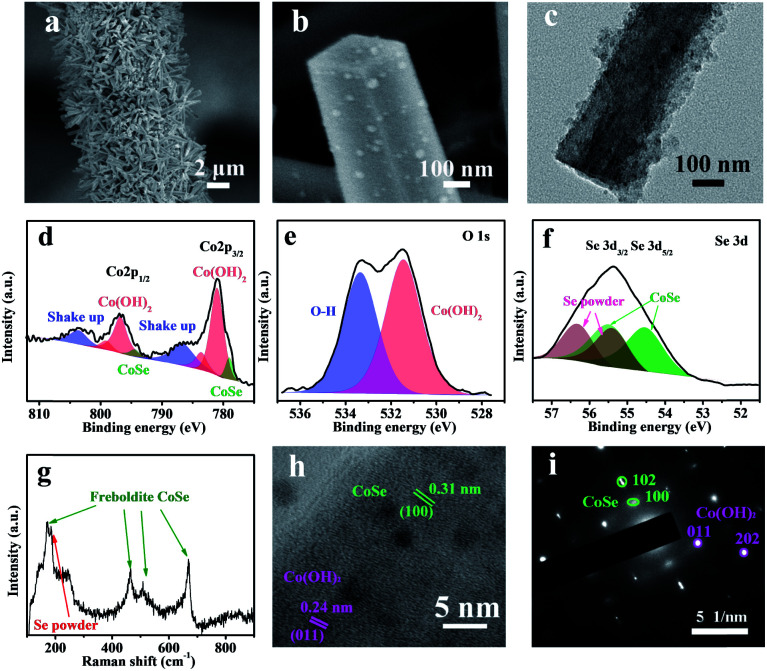
Structural, morphological and composition characterization for CC@Co(OH)_2_@CoSe nanorods. (a) Low-magnification and (b) high-magnification SEM images of CC@Co(OH)_2_@CoSe nanorods. (c) TEM image of an individual Co(OH)_2_CoSe nanorod. (d) High resolution Co 2p, (e) O 1s and (f) Se 3d XPS spectra. The binding energy obtained with reference to C 1s at 284.8 eV. (g) Raman spectra. (h) HRTEM image and (i) SAED pattern.

The chemical composition and bonding states were further investigated by X-ray photoelectron spectroscopy (XPS). The survey XPS spectrum in Fig. S5† shows that the CC@Co(OH)_2_@CoSe is composed of Co, O, C and Se. The high resolution Co 2p spectrum shows four peaks of Co 2p_3/2_ identified as Co–Se at 779 eV, Co–OH at 781 and 783.7 eV, and a Co^2+^ shake up peak at 786.6 eV (Fig. 2d). Another four peaks of Co 2p_1/2_ are identified as Co–Se at 794.4 eV, Co–OH at 796.9 and 799.3 eV, and a Co^2+^ shake up peak at 803.7 eV^33,52^ with energy separation between Co 2p_3/2_ and Co 2p_1/2_ of about 15.5 eV. The O 1s spectrum in Fig. 2e shows two peaks at 531.5 and 533.4 eV corresponding to Co^2+^–O and O–H in Co(OH)_2_. For the Se 3d spectrum, the four peaks of Se 3d_5/2_ and 3d_3/2_ correspond Co–Se^53^ and residual Se–Se with an energy separation of about 0.86 eV (Fig. 2f). The XRD patterns in Fig. S6b† show typical Co(OH)_2_ diffraction peaks (PDF card 03-0443) without CoSe diffraction signals, which may be due to the low crystallinity of CoSe obtained by the electrochemical transformation. But the Raman spectra provide evidence to support the formation of CoSe, where the four peaks at 169, 465, 508 and 669 cm^−1^ are assigned to freboldite CoSe,^32^ and the peak at 185 cm^−1^ is associated with the residual amorphous selenium powder (Fig. 2g). Furthermore, the high-resolution TEM (HRTEM) image also shows the Co(OH)_2_@CoSe with interplanar spacings of 0.24 nm for the lattice plane of Co(OH)_2_ (011) and 0.31 nm for CoSe (110) (Fig. 2h). The corresponding selected area electron diffraction (SAED) pattern further confirms that the NRs are composed of Co(OH)_2_ and CoSe (Fig. 2i). The above results demonstrate that the Co-based NRs are totally transformed to Co(OH)_2_@CoSe during the *in situ* electrochemical process. The chemical reactions during the transformation to produce Co(OH)_2_@CoSe are shown in eqn (1)–(3).

1 SeO_2_ + H_2_O → H_2_SeO_3_

2 H_2_SeO_3_ + H_2_O + 6e^−^ → Se^2−^ + 4OH^−^

3 2Co^2+^ + Se^2−^ + 2OH^−^ → Co(OH)_2_ + CoSe

To probe the key factors determining the Co(OH)_2_@CoSe NR formation, we also prepared CC@Co(OH)_2_@CoSe-25 and CC@Co(OH)_2_@CoSe-95 at temperatures of 25 °C and 95 °C. It was harder to form CoSe and maintain the Co(OH)F phase at lower synthesis temperatures (Fig. S6e and S7c†), but at higher synthesis temperatures it was easy to break the nanorod structure and form the Se powder phase (Fig. S6f and S7d†). CC@CoSe-ED was then fabricated using an electrodeposition method and CC@CoSe-CS was obtained by a direct calcination selenization method for comparison. For the CC@CoSe-ED, the carbon cloth was covered by an amorphous CoSe film (Fig. S6c and S7a†). For the CC@CoSe-CS, the vertical nanorod arrays were destroyed after the calcination treatment and were replaced by the CoSe_2_ particles (Fig. S6d and S7b†).

**Fig. 3:**
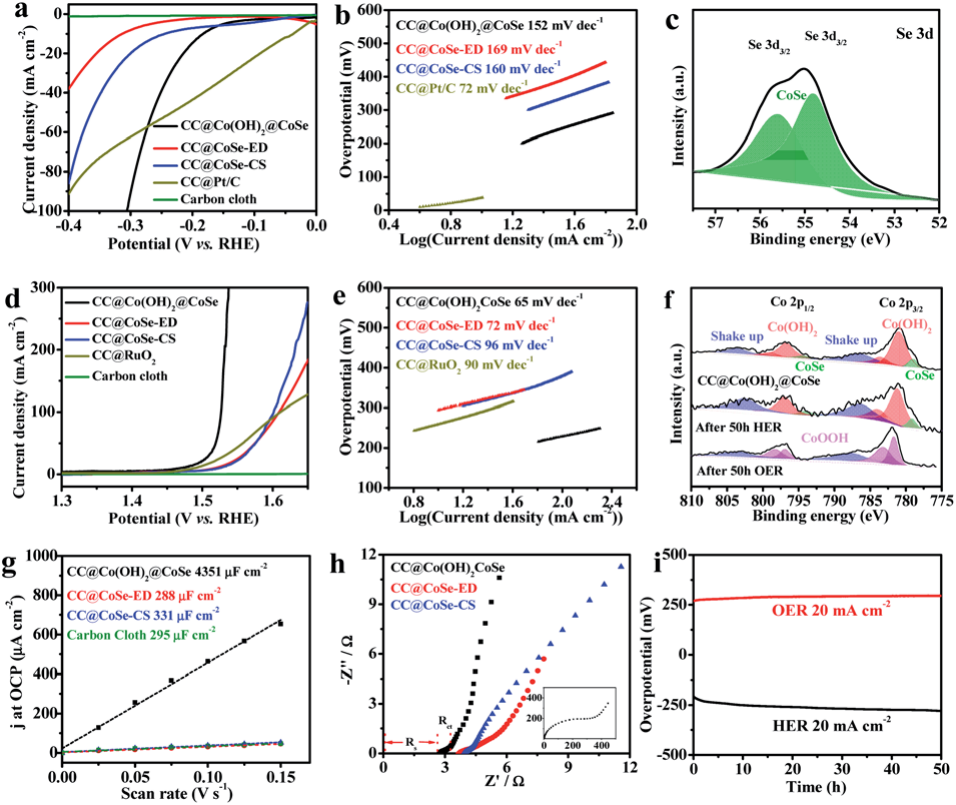
Electrocatalytic HER and OER performances in 1 M KOH (pH = 14). (a) HER polarization curves and (b) Tafel plots for CC@Co(OH)_2_@CoSe, CC@CoSe-ED, CC@CoSe-CS and CC@Pt/C. (c) Se 3d XPS spectrum of CC@Co(OH)_2_@CoSe after 50 h of the HER. (d) OER polarization curves and (e) Tafel plots for CC@Co(OH)_2_@CoSe, CC@CoSe-ED, CC@CoSe-CS and CC@RuO_2_. (f) Co 2p XPS spectra of CC@Co(OH)_2_@CoSe after 50 h of the HER and OER. (g) Plots of the current density at OCP *vs.* the scan rate and (h) EIS spectra of CC@Co(OH)_2_@CoSe, CC@CoSe-ED, and CC@CoSe-CS (inset, EIS spectrum of carbon cloth). (i) Chronopotentiometric measurements for long-term stability of CC@Co(OH)_2_@CoSe.

The electrocatalytic HER and OER activities were evaluated using the polarization linear sweep voltammograms (LSVs) recorded in a 1 M KOH aqueous solution, where all currents were corrected with 95% *iR* compensation. The CC@Co(OH)_2_@CoSe NRs exhibit excellent activity for the HER with an overpotential of 208 mV to reach a current density of 20 mA cm^−2^. In comparison, the overpotential is only 53 mV for CC@Pt/C, 355 mV for CC@CoSe-ED and 300 mV for CC@CoSe-CS (Fig. 3a and S10a†). Moreover, a Tafel plot was constructed to present the HER catalytic kinetics (Fig. 3b), where a Tafel slope of 152 mV dec^−1^ was obtained for CC@Co(OH)_2_@CoSe NRs, smaller than the 169 mV dec^−1^ for CC@CoSe-ED and the 160 mV dec^−1^ for CC@CoSe-CS. Although the Tafel slope is higher than the 72 mV dec^−1^ for CC@Pt/C, the CC@Co(OH)_2_@CoSe NRs show a remarkably high activity at large current density and an overpotential of only 314 mV at 100 mA cm^−2^, smaller than those of CC@Pt/C.

The CC@Co(OH)_2_@CoSe NRs also exhibit excellent electrocatalytic activity for the OER. Overpotentials of 297 mV and 303 mV are needed to achieve current densities of 100 mA cm^−2^ and 200 mA cm^−2^ (Fig. 3d and S10b†), in contrast to the 380 mV at 100 mA cm^−2^ for RuO_2_. Even at a current density of 20 mA cm^−2^, the overpotential is only 268 mV for CC@Co(OH)_2_@CoSe NRs, while it is 312 mV for CC@CoSe-ED, 316 mV for CC@CoSe-CS, 421 mV for CC@Co(OH)_2_ and 285 mV for CC@RuO_2_. Correspondingly, the Tafel slope of CC@Co(OH)_2_@CoSe NRs is 65 mV dec^−1^, smaller than the 72 mV dec^−1^ for CC@CoSe-ED, 96 mV dec^−1^ for CC@CoSe-CS and 90 mV dec^−1^ for CC@RuO_2_ (Fig. 3e). The effects of the electro-transformation reaction temperature and morphology on electrocatalytic activity were then investigated (Fig. S8†). The CC@Co(OH)_2_@CoSe NRs show superior performances for both the HER and OER compared to CC@Co(OH)_2_@CoSe-25 and CC@Co(OH)_2_@CoSe-95. The Co(OH)_2_@CoSe with broken NW morphology exhibits poor activities for both the HER and OER (Fig. S9†).

To investigate the surface active sites of CC@Co(OH)_2_@CoSe NRs during the electrocatalytic water splitting process, X-ray photoelectron spectroscopy (XPS) was conducted. Fig. 3c shows that only Co–Se bonds exist in the Se 3d spectrum after 50 h of the HER, which indicates that the residual Se powder dissolved in the alkaline medium during the HER process. But there is no difference in the Co 2p_3/2_ and Co 2p_1/2_ spectra for the as-prepared CC@Co(OH)_2_@CoSe NRs and the NRs after 50 h of the HER (Fig. 3f). After the OER process, the Co–Se bond disappeared and the Co 2p spectrum of Co(OH)_2_ shifts to higher binding energy related to CoOOH, indicating that the Co(OH)_2_@CoSe NRs undergo a second *in situ* phase transformation during the OER. The O 1s spectrum in Fig. S11† shifts to a lower binding energy further supporting the conversion into CoOOH. Generally, this oxidation transformation is very common in CoSe-based OER catalysts^54,55^ and CoOOH is considered the true active site for water oxidation. The SEM images show that the NR morphology still remains after 50 h of the HER process (Fig. S13a†), but it is replaced by hexagonal nanosheets after 50 h of the OER process (Fig. S13b†). The XRD pattern of CC@Co(OH)_2_@CoSe after 50 h of the OER also exhibits a typical hexagonal CoOOH phase (Fig. S14†).

In order to further understand the excellent activity, the electrochemically active surface area (ECSA) was calculated by measuring the scan-rate dependent cyclic voltammograms (CVs) in a 1 M KOH solution. The electrochemical double-layer capacitance (*C*_dl_) value of CC@Co(OH)_2_@CoSe NRs is 4351 μF cm^−2^, higher than the 295 μF cm^−2^ of carbon cloth, 288 μF cm^−2^ of CC@CoSe-ED and 331 μF cm^−2^ of CC@CoSe-CS (Fig. 3g and S12†). The roughness factor of CC@Co(OH)_2_@CoSe NRs was calculated to be 14.7, proving that the vertically oriented NR morphology can provide a more exposed active surface than the electrodeposition and calcination selenization methods. Electrochemical impedance spectroscopy (EIS) analysis was performed to gain an insight into the electron and mass transfer during the electrocatalytic process, where the series resistance (*R*_s_) in the Nyquist plot reflects the resistance of the electrolyte solution and the charge-transfer resistance (*R*_ct_) indicates the resistance of charge transfer in the catalyst. The CC@Co(OH)_2_@CoSe has the smallest *R*_s_ and *R*_ct_, which indicates good electrical contact and fast charge transfer properties (Fig. 3h). Moreover, the stability results present an increasing overpotential for the HER changing from 208 mV to 270 mV and for the OER from 268 mV to 300 mV over 50 h (Fig. 3i).

**Fig. 4:**
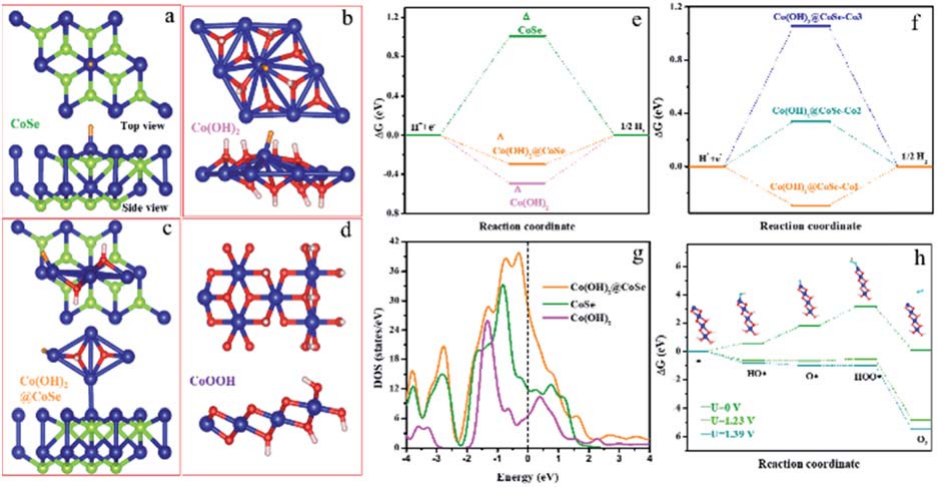
Co–OH active sites of CC@Co(OH)_2_@CoSe NRs during the HER and OER. DFT calculated structure models (upper: top view, bottom: side view) of (a) pure CoSe, (b) pure Co(OH)_2_, (c) Co(OH)_2_@CoSe and (d) CoOOH. (e) H adsorption free energy profiles of pure CoSe, pure Co(OH)_2_ and Co(OH)_2_@CoSe. (f) H adsorption free energy profiles of Co(OH)_2_@CoSe at different adsorption sites. (g) Density of states (DOS) of pure CoSe, pure Co(OH)_2_ and Co(OH)_2_@CoSe. (h) Energy diagram for the OER pathway of CoOOH (012). White, red, blue and green spheres represent H, O, Co and Se atoms, respectively. Δ represents adsorption sites in the HER pathway.

To deeply understand the catalytically active sites of the Co(OH)_2_@CoSe NRs, the key reaction coordinate in the alkaline HER and OER on the Co(OH)_2_@CoSe was investigated by DFT calculations. Three structural models of pure CoSe, pure Co(OH)_2_, and Co(OH)_2_@CoSe were constructed for the HER (Fig. 4a–c). Generally, three states can be observed during the HER process: an initial H^+^ state, the adsorption of an intermediate H*, and the final product of 1/2 H_2_. The Gibbs free energy of intermediate H* adsorption, Δ*G*_H*_, is regard as a major descriptor of HER activity for catalysts, and the Gibbs energy change of |Δ*G*_H*_| is as small as zero represents a highly efficient HER catalyst. A large positive Δ*G*_H*_ of 1.00 eV for pure CoSe means that it has very weak H* adsorption, while a large negative Δ*G*_H*_ of −0.49 eV for pure Co(OH)_2_ indicates that H* adsorption is too strong to desorb the final H_2_ product (Fig. 4e). For Co(OH)_2_@CoSe, Co-1 (Co–OH site at Co(OH)_2_), Co-2 (Co–Co at CoSe), and Co-3 (octahedral coordinated Co–Se at CoSe) represent three different Co sites (Fig. S15†), and the |Δ*G*_H*_| values for these sites are 0.29, 0.34 and 1.05, respectively (Fig. 4f). Thus, the Co–OH site is the most favourable site for H* adsorption and desorption for the HER. The |Δ*G*_H*_| value of the Co–OH active site is much smaller than that of pure-CoSe and pure-Co(OH)_2_, facilitating H_2_ desorption. Additionally, the density of states (DOS) results show that Co(OH)_2_@CoSe has higher DOS close to the Fermi level (Fig. 4g), which implies a higher electron transfer capability for an enhanced chemical activity. Therefore, chemical coupling of Co(OH)_2_@CoSe can regulate the adsorption and desorption energy of Co–OH active sites to efficiently facilitate the HER kinetics.

Then, we investigated the tailoring of CoOOH (012) on the Gibbs free energy along the OER pathway for *OH, *O, and *OOH (Fig. 4d). The most favourable site of CoOOH is the unsaturated coordinated Co–OH site that can adsorb the above three intermediates, with Δ*G* values of 0.59 eV, 1.78 eV, and 3.17 eV (Fig. 4h). The step from *O to *OOH is the rate-determining step (RDS) due to its largest energy barrier of 1.39 eV. When increasing the potential (*U*) to 1.23 V, its Δ*G* values for *OH, *O, and *OOH are −0.64 eV, −0.67 eV, and −0.51 eV. The RDS is almost equal to zero until *U* increases to 1.39 eV. The *in situ* converted CoOOH shows a desired OER activity with the Co–OH site serving as the reactive active site for tailoring the RDS energy of these oxygenated intermediates in the alkaline environment, which facilitates the overall OER kinetics.

**Fig. 5:**
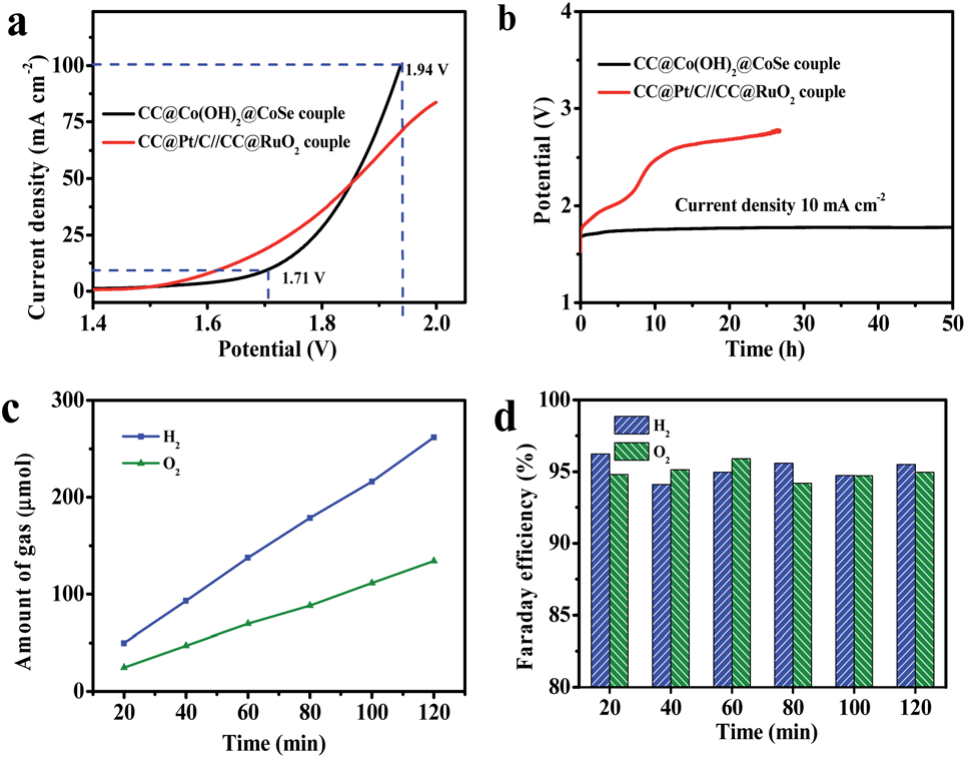
Electrolytic cell performance for overall water splitting. (a) Polarization curves and (b) electrolytic stabilities at 10 mA cm^−2^ for around 50 h for CC@Co(OH)_2_@CoSe NRs and the CC@Pt/C//CC@RuO_2_ cell in the two-electrode configuration. (c) Experimentally measured amount of H_2_ and O_2_. (d) Faraday efficiencies of the HER and OER *versus* time for the CC@Co(OH)_2_@CoSe NR cell.

Accordingly, encouraged by the excellent OER and HER performances of CC@Co(OH)_2_@CoSe NRs at a large current density, we assembled an overall water splitting electrolytic cell in a 1 M KOH solution with the NRs as both the anode and the cathode (Fig. 5a). For comparison, a CC@Pt/C//CC@RuO_2_ cell was also examined under the same conditions with CC@RuO_2_ as the anode and CC@Pt/C as the cathode. The CC@Co(OH)_2_@CoSe cell needed a potential of 1.71 V to realize overall water splitting at a current density of 10 mA cm^−2^, slightly higher than that of the CC@Pt/C//CC@RuO_2_ cell (10 mA cm^−2^ at 1.64 V). But, at high current density, the CC@Co(OH)_2_@CoSe cell showed a better performance, where it presented 100 mA cm^−2^ at 1.94 V in comparison with the 70 mA cm^−2^ at 1.94 V for the CC@Pt/C//CC@RuO_2_ cell. Moreover, the CC@Co(OH)_2_@CoSe cell exhibited an excellent electrolytic stability. The operating voltage for the CC@Co(OH)_2_@CoSe cell required to sustain a current density of 10 mA cm^−2^ only underwent a 67 mV increase after 50 h (Fig. 5b). However, the CC@Pt/C//CC@RuO_2_ cell exhibited a remarkable potential increase in a very short period, which may be due to the poor stability of RuO_2_ in the alkaline medium. The Faraday efficiency value was calculated from the experimentally measured ratio of H_2_ to O_2_ (2 : 1) (Fig. 5c) and it remains above 94% during 2 h of electrolysis (Fig. 5d).

## Conclusion

In summary, we have developed an *in situ* electrochemical transformation method to fabricate Co(OH)_2_@CoSe nanorods as a bifunctional electrocatalyst. The obtained Co(OH)_2_@CoSe NRs exhibit an excellent electrocatalytic activity and stability for the simultaneous HER and OER. The chemical coupling Co–OH active site between Co(OH)_2_ and CoSe can optimize the adsorption and desorption energy for hydrogen to efficiently facilitate the HER. The CoOOH converted by a second electro-transformation during the OER acts as the real active sites for the OER. These merits make the practical electrolytic cell exhibit high overall water splitting efficiency at a large current density. This work provides a novel electro-synthesis technique to develop noble metal-free chemical coupling active sites to regulate electrocatalysts towards real-world water splitting electrolytic cell applications.

## Author contributions

Y. W. and Y. T. Y. contributed equally. J. H. L., L. M. D., and C. W. H. planned and supervised the research. J. H. L., Y. W., and Y. T. Y. co-wrote the manuscript. Y. W. and Y. T. Y. designed and performed most of the experiments. X. W. conducted the simulations. P. H. L. and H. Y. S. participated in some of the experiments. T. E. L. and Q. F. Z. conducted XPS and SEM analysis. H. Y. L. and J. H. performed electrochemical measurements. All the authors discussed the results and commented on the manuscript.

## Conflicts of interest

There are no conflicts to declare.

## Supplementary Material

NA-002-C9NA00725C-s001
