# Supplementary material for: Electro-synthesized Co(OH)2@CoSe with Co–OH active sites for overall water splitting electrocatalysis
Source: Nanoscale Adv. 2020 Jan 6;2(2):792–7. doi: 10.1039/c9na00725c (PMC9417052; doi:10.1039/c9na00725c)
Supplement: NA-002-C9NA00725C-s001 [file NA-002-C9NA00725C-s001.pdf]

Electronic Supplementary Material (ESI) for Nanoscale Advances.
This journal is © The Royal Society of Chemistry 2020

Supporting Information

Electro-synthesized $Co(OH)_2$@CoSe with Co-OH Active Sites for Overall Water Splitting Electrocatalysis

Yin Wang[a, ‡], *Yutong Yang*[a, ‡], *Xia Wang*[a], *Peihe Li*[a], *Hongyang Shao*[a], *Tianen Li*[a], *Haiyang Liu*[a], *Qingfu Zheng*[a], *Jing Hu*[a], *Limei Duan*[*a], *Changwen Hu*[*b] *and Jinghai Liu*[*a]

[a]Inner Mongolia Key Laboratory of Carbon Nanomaterials, College of Chemistry and Chemical Engineering, Nano Innovation Institute, Inner Mongolia University for Nationalities, Tongliao 028000, China.

[b]Key Laboratory of Cluster Science, Ministry of Education of China, Beijing Key Laboratory of Photoelectronic/Electrophotonic Conversion Materials, School of Chemistry and Chemical Engineering, Beijing Institute of Technology, Beijing 100081, China.

[‡]These two authors contributed equally.

*Corresponding Authors: jhliu2008@sinano.ac.cn; duanlmxie@126.com; cwhu@bit.edu.cn

Experimental

Materials: All chemicals were of analytical grade and were used without further purification. Cobalt nitrate hexahydrate ($Co(NO_3)_2 \cdot 6H_2O$), cobalt acetate tetrahydrate ($CoC_4H_{14}O_8 \cdot 4H_2O$), ammonium fluoride ($NH_4F$), urea ($CO(NH_2)_2$), selenium oxide ($SeO_2$), selenium powder (Se), potassium hydroxide (KOH), sulphuric acid (H_2SO_4), nitric acid (HNO_3), benzene (C_6H_6), ethanol (CH_3OH) and acetone (CH_3COCH_3) were purchased from Sinopharm Chemical Reagent Co. Ltd. Before using, carbon cloth (CC, WOS1002 CeTech Co. Ltd.) was ultrasonically cleaned with acetone, ethanol, deionized water for 30 min and nitric acid for 4 h.

Preparation of CC@Co-based nanorods/nanowires: For CC@Co-based nanorods (NRs) synthesis, 4 mmol of $CoC_4H_{14}O_8$ (cobalt acetate), 8 mmol of NH_4F (ammonium fluoride) and 20 mmol of $CO(NH_2)_2$ (urea) were dissolved in 105 mL mixed solvent (deionized water: ethanol = 2:1) under magnetically stirring for 30 min to form a homogeneous solution. The solution was transferred into 50 mL Teflon-lined stainless steel autoclave. Then, a piece of the pre-treated CC (ca. 2 × 2 cm) was vertically immersed in above solution. Hydrothermal synthesis was carried out at 100 °C for 10 h. After cooling to room temperature naturally, the film was ultrasonically cleaned for 5 min with deionized water, dried at 60 °C for 2 h. For CC@Co-based nanowires (NWs) synthesis, the reaction mixture was 105 mL deionized water containing 3 mmol $Co(NO_3)_2$ (cobalt nitrate), 3 mmol NH_4F and 15 mmol of $CO(NH_2)_2$, then the hydrothermal temperature was changed to 120 °C, other conditions were the same as above.

In-situ electrochemcial transformation to fabricate $Co(OH)_2$@CoSe NRs: CC@Co-based NRs in situ transform to CC@$Co(OH)_2$@CoSe NRs was realized in a mild electrochemical method. The electro-transformation was carried out in a three-electrode consisting of a platinum sheet (2 × 1 cm, counter electrode), a saturated calomel electrode (SCE, reference electrode), and the as-prepared CC@Co-based NRs (working electrode, ca. 2 × 1 cm) connected to an electrochemical analyzer (CHI 660E Instruments). The electrodes were immersed in the 80 mL electrolyte (10 mM SeO_2

with 50 mM KCl). During the experiment, the electrolyte was kept at constant temperature (25, 60, 95 °C) in water bath. The electrochemical transformation was carried out at –0.7 V vs. SCE for 40 min. Then the as-prepared samples were ultrasonically cleaned for 2 min with benzene, ethanol and deionized water to remove the adsorbed selenium and salt, dried at room temperature.

Preparation of CC@CoSe-ED (electrodeposition) and CC@CoSe-CS (calcination selenization): For comparison, the CC@CoSe-ED was prepared by electrodeposition. The electrodeposition was carried out in a three-electrode system, a Pt sheet as the counter electrode, a SCE as the reference electrode, a clean carbon cloth (ca. 2 × 1 cm) as the working electrode, 80 mL solution of 10 mM $CoCl_2$, 10 mM SeO_2 and 50 mM KCl as the electrolyte. The electrodeposition was carried out by chronoamperometry method at -0.7 vs. SCE for 40 min. After washed by benzene, ethanol and deionized water, the sample was dried at room temperature and named as CC@CoSe-ED. The CC@CoSe-CS was prepared by calcination selenization method. A piece of CC@Co-based NRs (2 × 1 cm) with 0.5 g of Se powder were placed in a tube furnace, while the Se powders were at the upstream side. The sample was heated by 450 °C for 30 min under 100 sccm Ar flow, the heating rate was 20 °C min^{-1}. The sample was named as CC@CoSe-CS.

Fabrication of CC@Co(OH)$_2$, CC@Pt/C and CC@RuO$_2$ electrodes: 5 mg of $Co(OH)_2$, 20% Pt/C or RuO_2 were dispersed in 1 mL of solution (containing 700 μL H_2O, 270 μL ethanol and 30 μL 5 wt% Nafion aqueous), then the solution was ultrasonically treated for 1 h to obtain a homogeneous dispersion. 260 μL of dispersion was dropped onto 1 cm^2 of clean carbon cloth to obtain a catalyst loading of 1.3 mg cm^{-2}.

Characterizations: The morphology and microstructure of the samples were characterized by field emission scanning electronic microscope (SEM, JSM-7500F), and transmission electron microscope (TEM, H-8100). Raman spectra were recorded on Raman system (LabRM Aramis) with a 532 nm laser. X-ray photoelectron spectroscopy (XPS) was conducted on an ESCALAB 250 spectrometer using Al Kα

radiation as the X-ray source (1486.7 eV) with the pass energy of 30 eV. Powder X-ray diffraction (XRD) data were recorded on a SmartLab 9kW (Rigaku) equipped with graphite-monochromatized Cu Ka radiation (λ = 0.1541 nm; scan speed of 4 min^{-1}; 2θ = 10-80°) at room temperature.

Electrochemical characterizations: All of the electrochemical characterizations were measured on a CHI 660E electrochemical workstation. A platinum sheet as counter electrode, a Ag/AgCl/saturated KCl as reference electrode, the as-prepared electrocatalysts as working electrode and 1 M KOH (pH = 14) aqueous solution as electrolyte. Before HER and OER tests, 20 cycles of cyclic voltammetry (CV) were performed, the scan rate was 100 mV s^{-1}. Linear sweep voltammetry (LSV) was carried out at 5 mV s^{-1} (HER) and 1 mV s^{-1} (OER) for the polarization curves and the Tafel plot was calculated by LSV data. All polarization curves were corrected with 95% iR-compensation. The potential and overpotential for HER/OER were calculated according to the following equation:

$$E\ (\text{potential, V}) = E\ (\textit{vs.}\ \text{Ag/AgCl}) + 0.197 + 0.0592 \cdot \text{pH}\ [\textit{vs.}\ \text{RHE}] \quad (1)$$

$$\text{Overpotential}\ (\eta, \text{OER, V}) = E\ (\text{potential, V}) - 1.23\ \text{V} \quad (2)$$

$$\text{Overpotential}\ (\eta, \text{HER, V}) = -E\ (\text{potential, V}) \quad (3)$$

The Tafel slope was calculated according to the Tafel equation as follows:

$$\eta = a + b \log j \quad (4)$$

where b denotes the Tafel slop, a is the overpotential at a current density of 1 mA cm^{-2}, and j denotes the current density under the given overpotential (η) for each HER and OER.

The electrochemically active surface area (ECSA) were estimated by double layer capacitance measurement. A series of cyclic voltammetry were performed across ± 20

mV at the open circuit potential (OCP) for different samples at 25, 50, 75, 100, 125 and 150 mV s^{-1} scan rates. The anodic current densities at the OCP were plotted against the scan rates. The slope was recorded as the double layer capacitance (C_{dl}). The roughness factor is calculated by the following equation:

$$Roughness\ fator = \frac{C_{dl}\ (sample)}{C_{dl}\ (carbon\ cloth\ substrate)} \quad (5)$$

The electrochemical impedances were measured in 1 M KOH solution. The applied potential was 1.423 V *vs.* RHE. The amplitude of sinusoidal wave was 10 mV, and the frequency scan range was from 100 kHz to 1 Hz. The chronopotentiometric (CP) measurements were carried out to examine the stability of $Co(OH)_2$@CoSe for both HER and OER.

Computational Method: First principles calculations based on density functional theory (DFT) were performed by using Vienna ab initio simulation package (VASP). The exchange-correlation energy of the projector augmented wave (PAW) potentials and the generalized gradient approximation was described by Perdew, Burke, and Grimme (GGA-PBE). The plane-wave kinetic energy cutoff was set to 400 eV. For structural relaxation, a 9 × 9 × 1 Monhorst-Pack k-point mesh was used, and the convergence criterion of 10^{-4} eV and 0.02 eVÅ-1 were chosen. A vacuum layer of 15 Å is applied to avoid the interaction between the neighboring slabs. The Gibbs free energy was calculated as follows: $\Delta G = \Delta E + \Delta ZPE - T\Delta S$, where ΔG, ΔE, ΔZPE and TΔS are the Gibbs free energy, total energy, zero point energy and entropic contributions from DFT calculations, respectively.

Electrolytic cell for overall water splitting to H_2 and O_2 and Faraday efficiency (FE): The overall water-splitting reaction was performed in a two-electrode configuration electrolytic cell using CC@$Co(OH)_2$@CoSe as both anode and cathode. The CP experiments were carried out as same as their HER and OER measurements. The relative evolution of H_2 and O_2 during each half reaction and overall reaction were tested by a gas chromatography (GC7920, Beijing Aulight) in a closed electrolytic cell.

The standard H_2 and O_2 gas were applied to obtain a calibration curves (**Fig. S16**). The amounts of H_2 and O_2 were calculated according to this calibration curve (**Fig. S17**). The Faraday efficiency was calculated by the numbers of electron transferred according the evolved gases as following equation:

$$FE(\%) = \frac{moles\ of\ products\ \times\ number\ of\ electrons\ needed)}{moles\ of\ electrons\ passed} \times 100\% \tag{6}$$

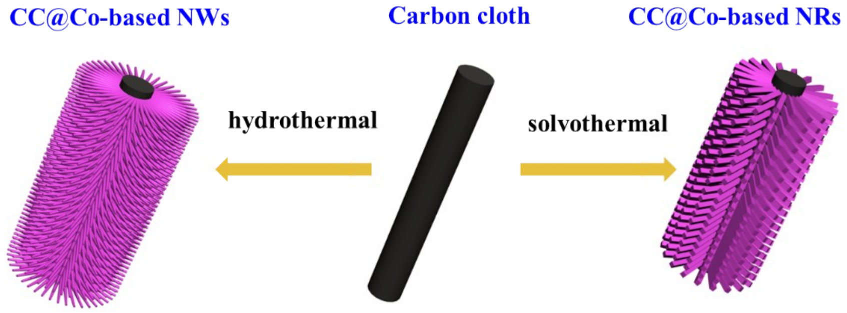

Figure S1. Schematic illustration of hydrothermal and solvothermal method to obtain the Co-based nanowires (NWs) and nanorods (NRs) templates.

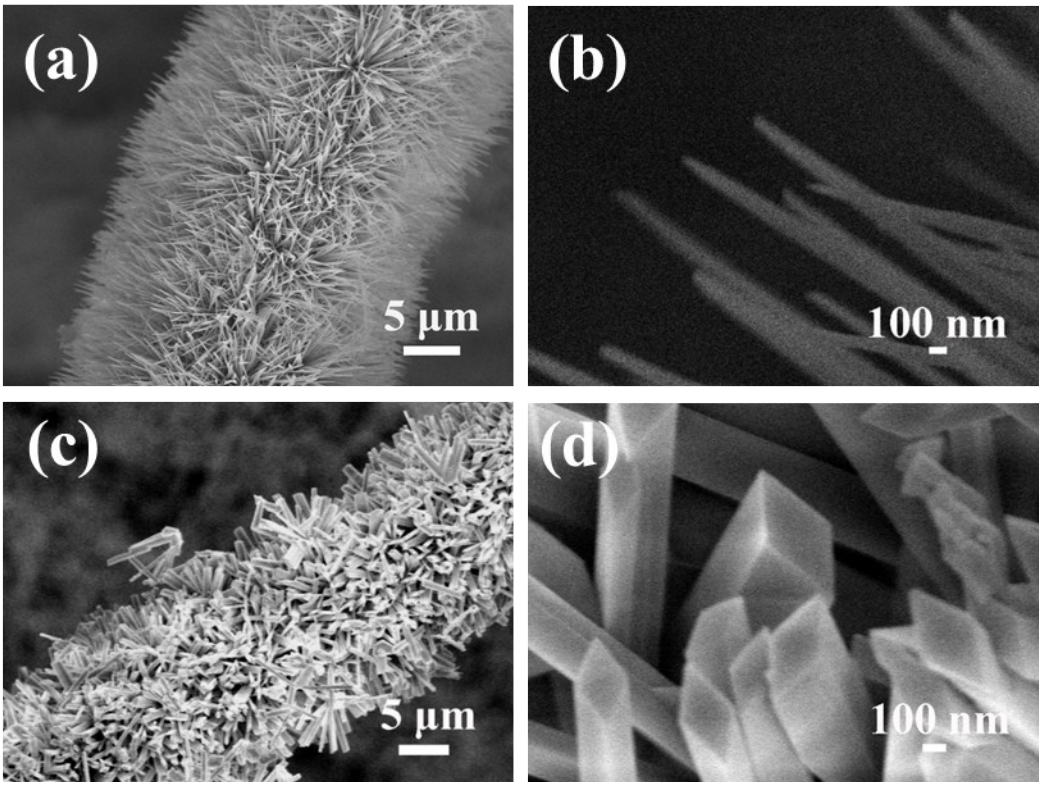

Figure S2. SEM images of **(a, b)** CC@Co-based NWs and **(c, d)** CC@Co-based NRs at different magnifications.

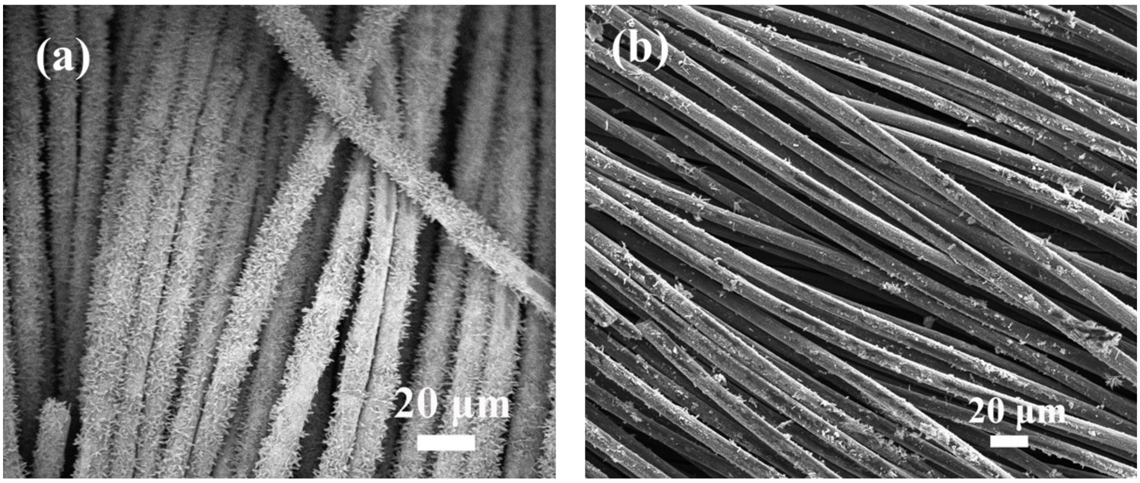

Figure S3. SEM image of **(a)** CC@Co-based NRs and **(b)** CC@Co-based NWs after electrochemical selenization.

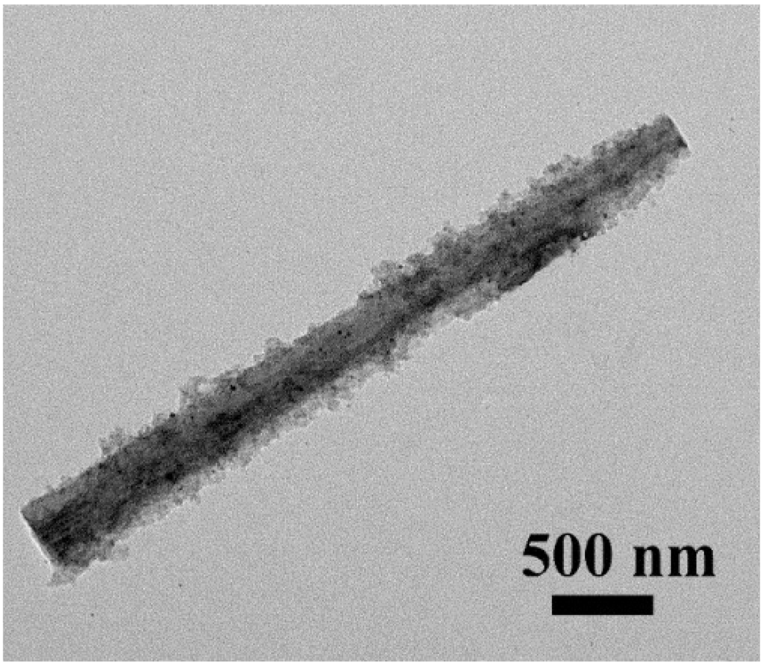

Figure S4. TEM image for one individual $Co(OH)_2$@CoSe nanorod.

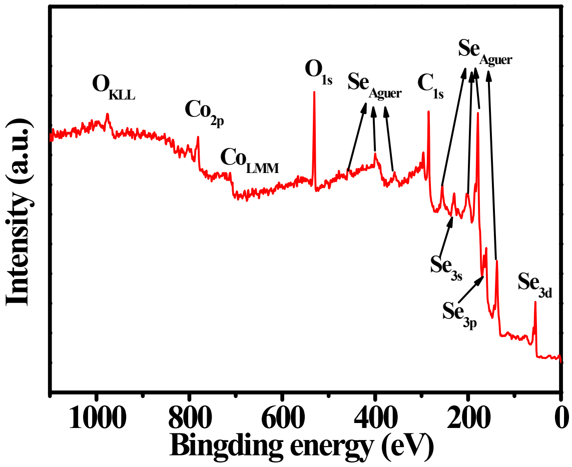

Figure S5. Survey XPS of CC@Co$(OH)_2$@CoSe. The binding energy was obtained with reference to the C1s at 284.8 eV.

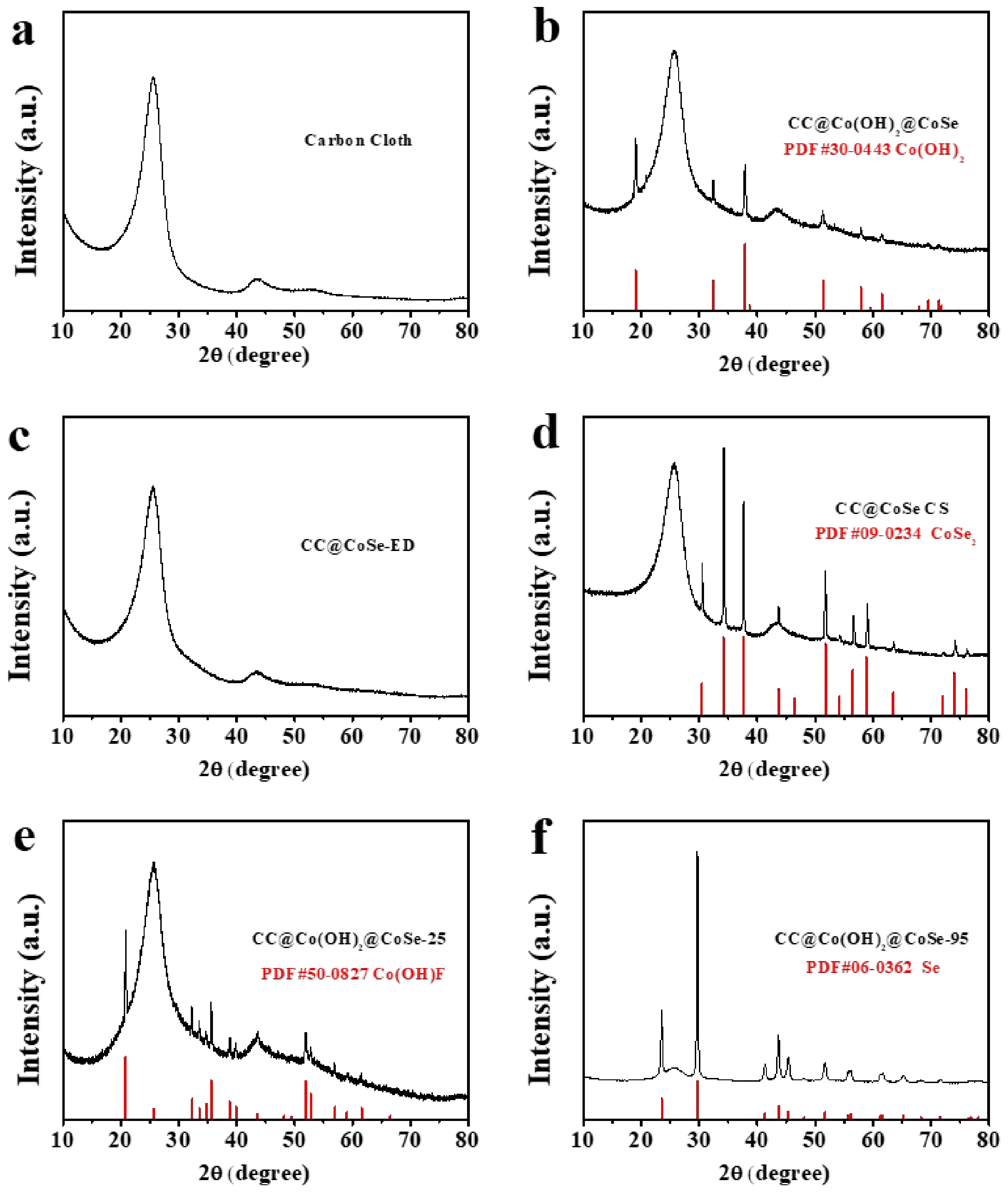

Figure S6 XRD patterns of (a) Carbon Cloth, (b) CC@Co(OH)$_2$@CoSe, (c) CC@CoSe-ED, (d) CC@CoSe-CS, (e) CC@Co(OH)$_2$@CoSe-25 and (f) CC@Co(OH)$_2$@CoSe-95.

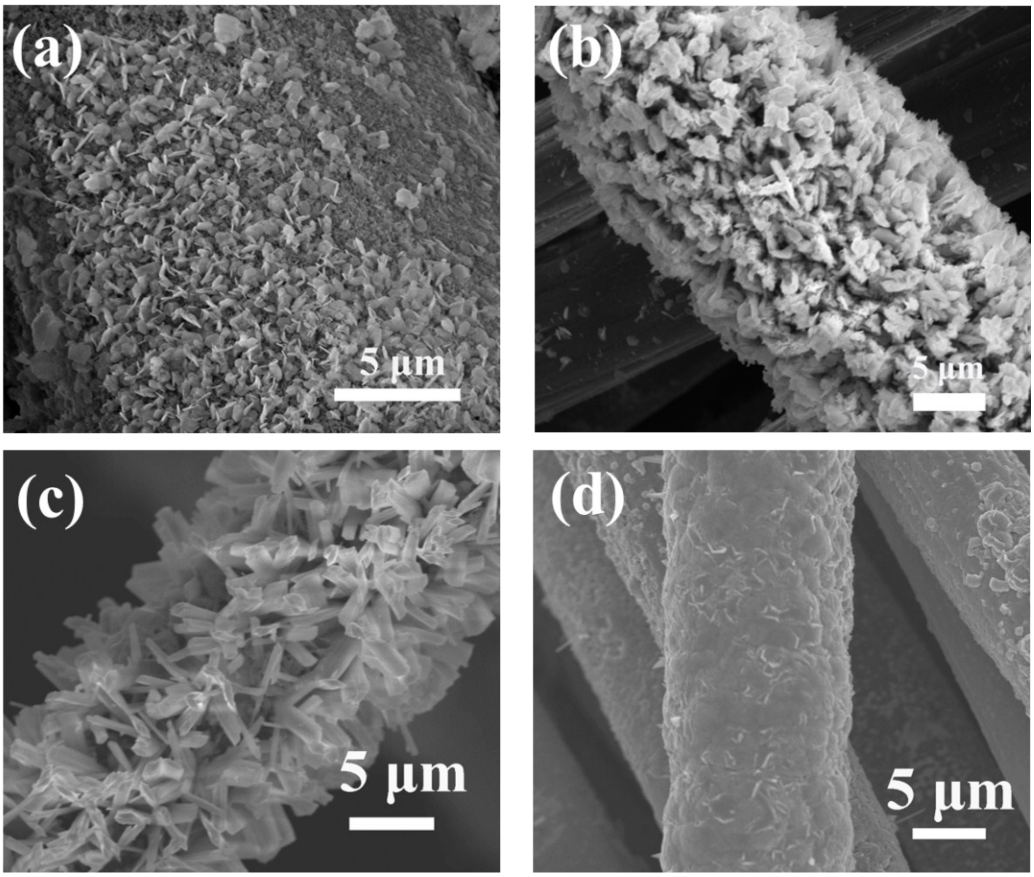

Figure S7. SEM images of (a) CC@CoSe-ED, (b) CC@CoSe-CS, (c) CC@Co$(OH)_2$@CoSe-25 and (d) CC@Co$(OH)_2$@CoSe-95.

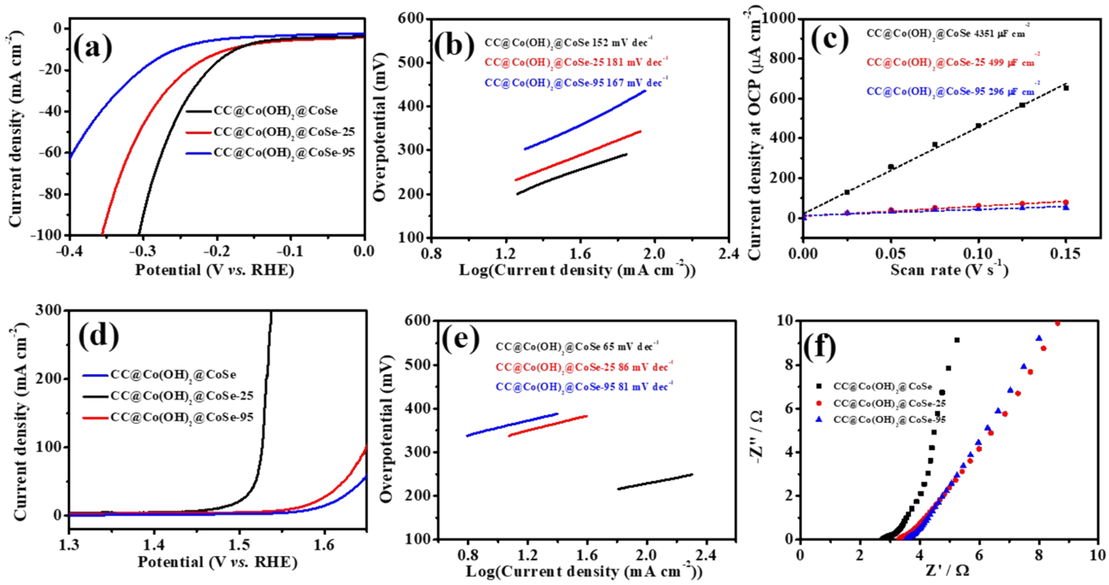

Figure S8. Polarization curves and Tafel plots for **(a, b)** HER and **(d, e)** OER, **(c)** Plots of the current density at OCP *vs.* the scan rate. **(f)** EIS of CC@$Co(OH)_2$@CoSe obtained by different temperatures.

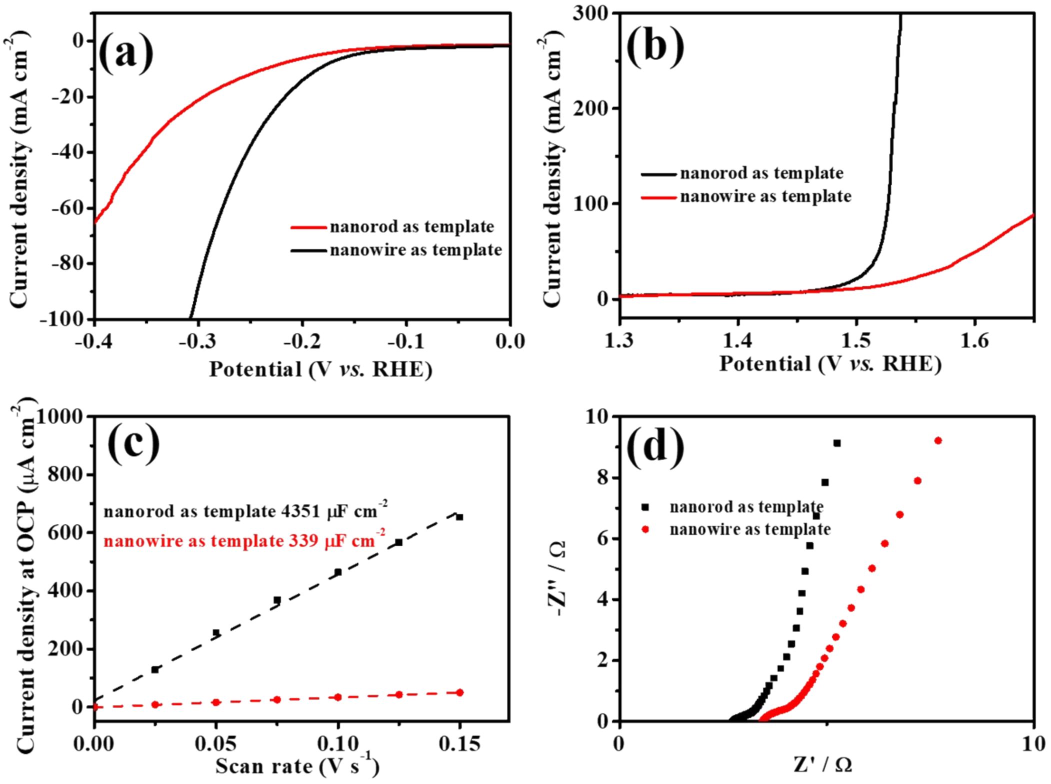

Figure S9. **(a)** HER polarization curves, **(b)** OER polarization curves, **(c)** Plots of the current density at OCP *vs.* the scan rate and **(d)** EIS of CC@$Co(OH)_2$@CoSe obtained from different templates.

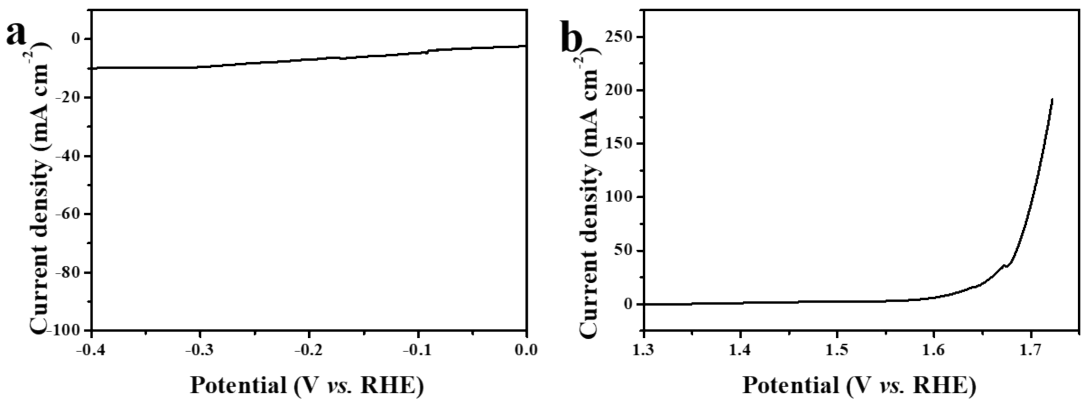

Figure S10. (a) HER and **(b)** OER polarization curves of CC@$Co(OH)_2$.

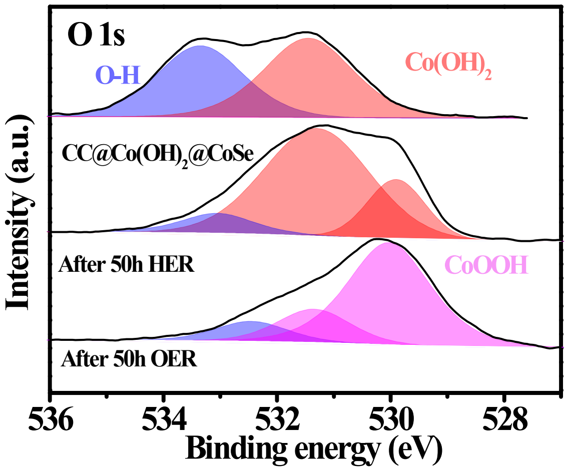

Figure S11. Comparison of O1s XPS spectra of as-prepared CC@Co(OH)$_2$@CoSe after 50 h HER and 50h OER. The binding energies were obtained with reference to the C1s at 284.8 eV.

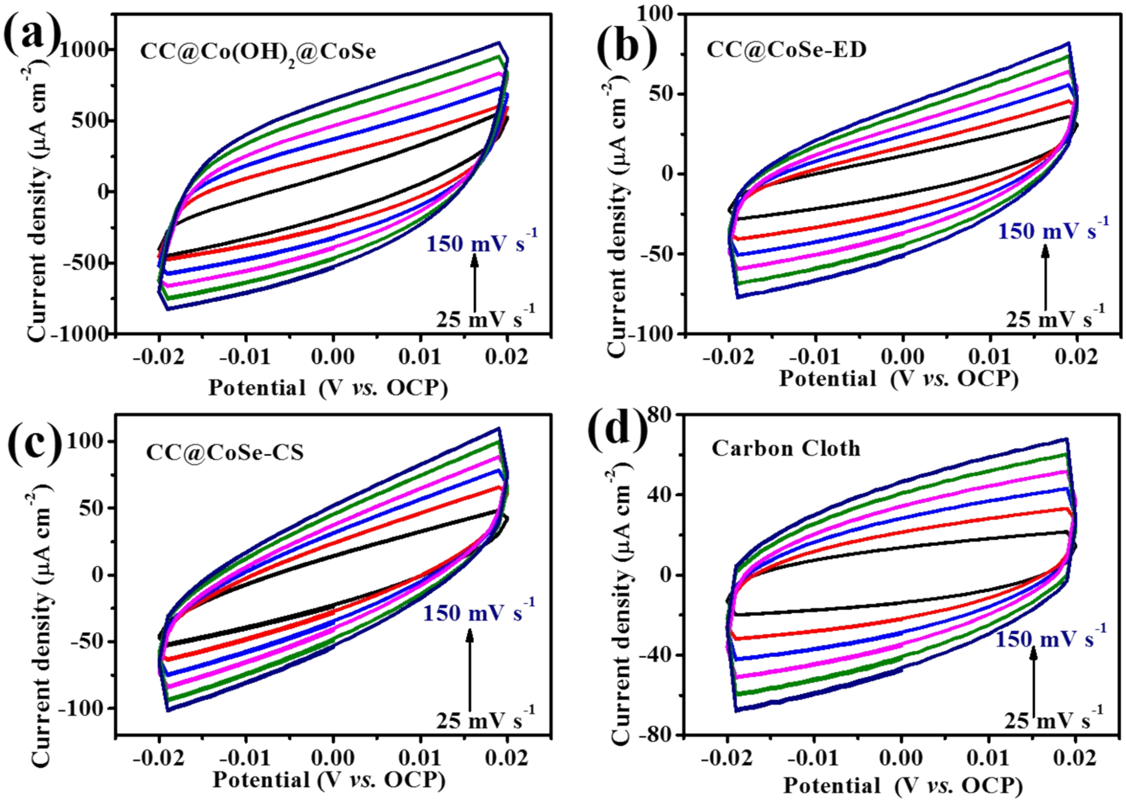

Figure S12. Cyclic voltammograms (CVs) tested at the potential range from 0.02 to 0.02 V vs. open circuit potential (OCP) with the scan rates increasing from 25 to 150 mV s^{-1} for **(a)** CC@Co$(OH)_2$@CoSe, **(b)** CC@CoSe-ED, **(c)** CC@CoSe-CS and **(d)** Carbon cloth.

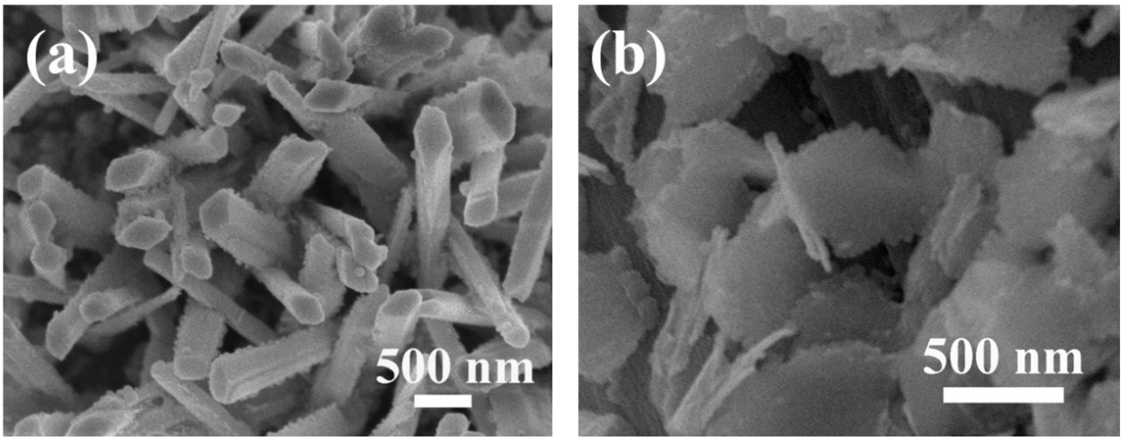

Figure S13. SEM images of $Co(OH)_2$@CoSe after **(a)** 50 h HER and **(b)** 50 h OER.

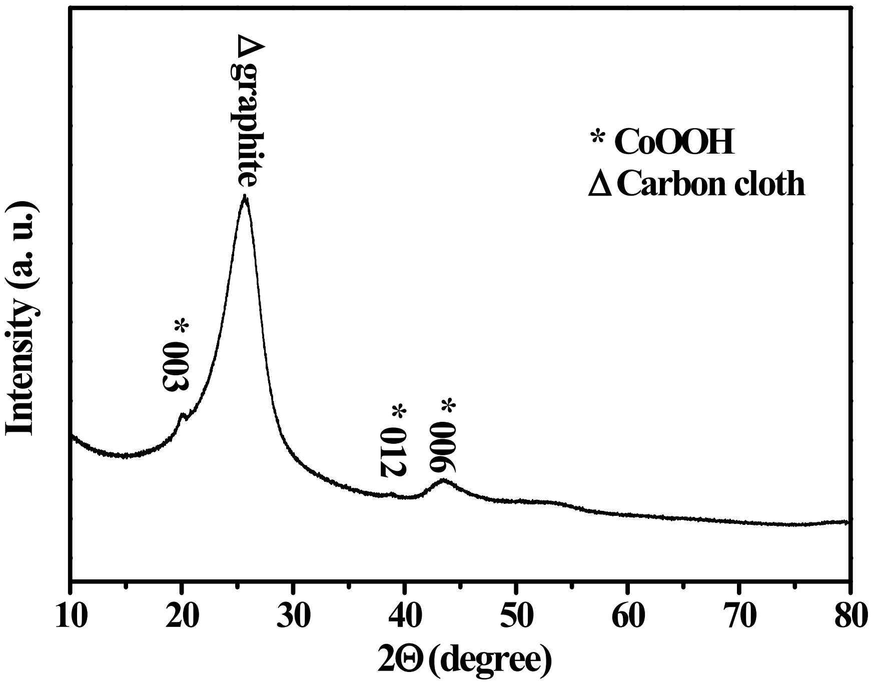

Figure S14 XRD pattern of CC@Co$(OH)_2$@CoSe after 50 h OER.

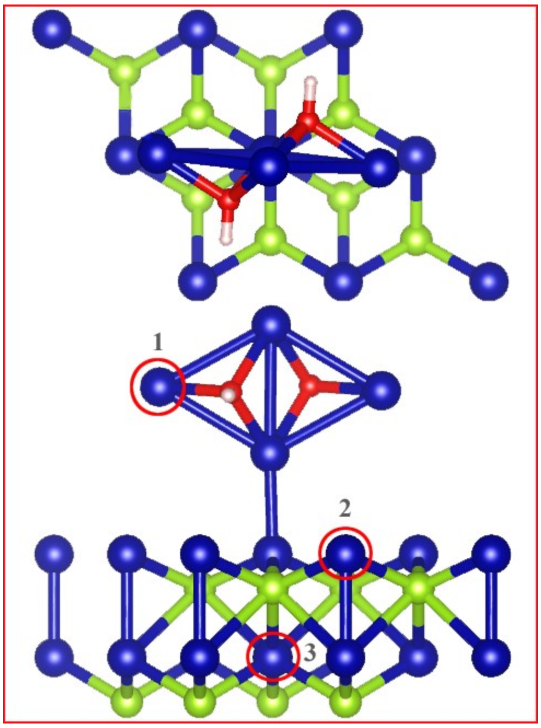

Figure S15. DFT calculated structures of $Co(OH)_2$@CoSe with different Co active sites, Co-1 (Co-OH site at Co(OH)2), Co-2 (Co-Co at CoSe), Co-3 (octahedral coordinated Co-Se at CoSe).

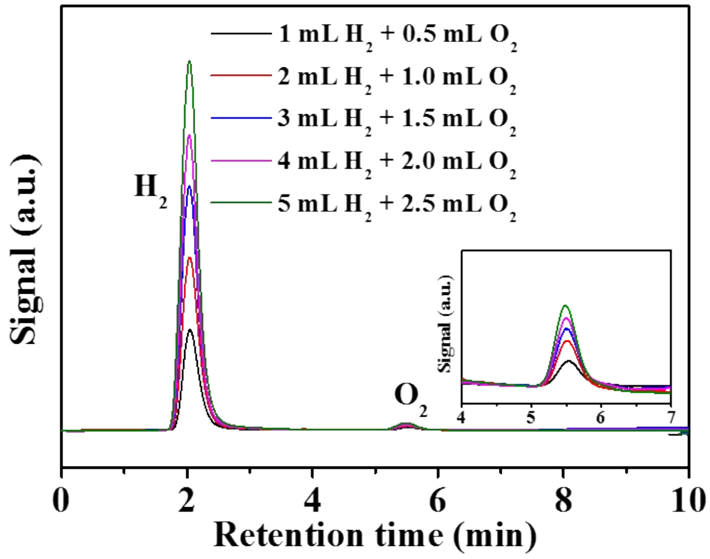

Figure S16. Gas chromatography signals for standard gas of H_2 and O_2 (inset).

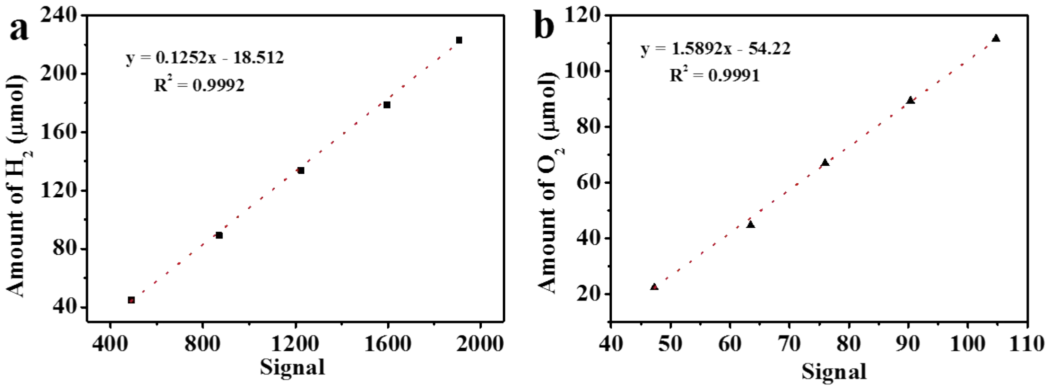

Figure S17. The calibration curves for amounts of **(a)** H_2 **(b)** and O_2 *vs.* gas chromatography signals.

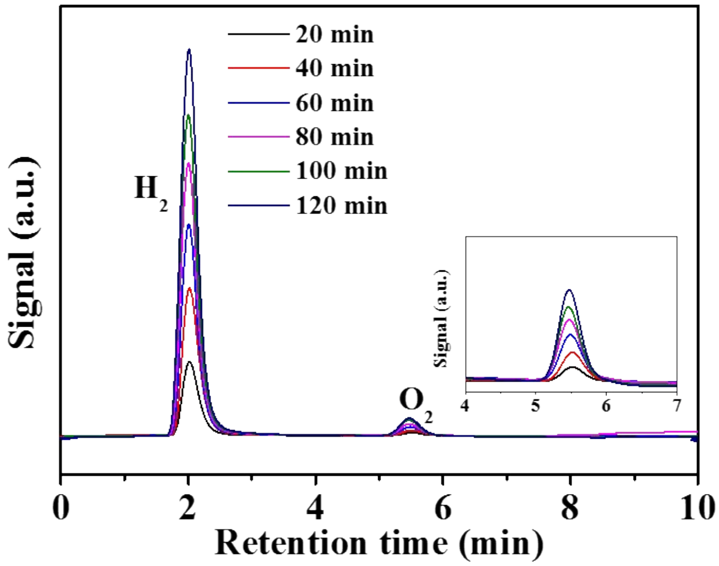

Figure S18. Gas chromatography signals for CC@Co(OH)$_2$@CoSe electrolytic cell.

Table S1. Comparison of reported $CoSe_x$-based electrocatalysts for overall water splitting.

Catalysts	Electrolytes	Properties	J/mA cm^{-2}	Overpotential/mV	Ref.
CC@$Co(OH)_2$@CoSe NRs	1M KOH	HER	20/100	208/314	This work
		OER	20/100/200	268/297/303	
		Water splitting	10/100	1.64 V/1.94 V	
Amorphous CoSe/Ti	1M KOH	HER	10	121	1
		OER	10	292	
		Water splitting	10	1.65 V	
EG/H-$Co_{0.85}Se$/NiFe-LDH	1M KOH	HER	10	260	2
		OER	150/250	270/280	
		Water splitting	20	1.71 V	
c-$CoSe_2$ NCs	1M KOH	HER	10	520	3
		OER	10	430	
		Water splitting	-	-	
CoSe/graphene/Ni mesh	1M KOH	HER	10	78	4
		OER	Onset	360	
		Water splitting	-	-	
$CoSe_2$/CF	1M KOH	HER	20	113	5
		OER	10	322	
		Water splitting	10	1.63 V	
Amorphous CoOx-CoSe/Ni	1M KOH	HER	10	90	6
		OER	100/500	300/380	

		Water splitting	20	1.66 V	
$Co(S_{0.71}Se_{0.29})_2$ /CC	1M KOH	HER	10	145	7
		OER	10	290	
		Water splitting	20	1.63 V	
EG/H-$Co_{0.85}Se$/P	1M KOH	HER	10	150	8
		OER	-	-	
		Water splitting	10	1.64 V	
Co0.85Se@NC	1M KOH	HER	10	230	9
		OER	10	320	
		Water splitting	10	1.76 V	
Se-(NiCo)S_x/$(OH)_x$ /NF	1M KOH	HER	10	103	10
		OER	10	155	
		Water splitting	10	1.6 V	
CoSe/Co_9Se_8/Co foil	1M KOH	HER	100	416	11
		OER	100	410	
		Water splitting	10	1.8 V (seawater)	

References

1 T. Liu, Q. Liu, A. M. Asiri, Y. Luo and X. Sun, *Chemical communications*, 2015, **51**, 16683-16686.

2 Y. Hou, M. R. Lohe, J. Zhang, S. Liu, X. Zhuang and X. Feng, *Energy Environ. Sci.*, 2016, **9**, 478-483.

3 I. H. Kwak, H. S. Im, D. M. Jang, Y. W. Kim, K. Park, Y. R. Lim, E. H. Cha and J. Park, *ACS Appl. Mater. Inter.*, 2016, **8**, 5327-5334.

4 X. Li, L. Zhang, M. Huang, S. Wang, X. Li and H. Zhu, *J. Mater. Chem. A*, 2016, **4**, 14789-14795.

5 C. Sun, Q. Dong, J. Yang, Z. Dai, J. Lin, P. Chen, W. Huang and X. Dong, *Nano Res.*, 2016, **9**, 2234-2243.

6 X. Xu, P. Du, Z. Chen and M. Huang, *J. Mater. Chem. A*, 2016, **4**, 10933-10939.

7 L. Fang, W. Li, Y. Guan, Y. Feng, H. Zhang, S. Wang and Y. Wang, *Adv. Funct. Mater.*, 2017, **27**, 1701008.

8 Y. Hou, M. Qiu, T. Zhang, X. Zhuang, C.-S. Kim, C. Yuan and X. Feng, *Adv. Mater.*, 2017, **29**, 1701589.

9 T. Meng, J. Qin, S. Wang, D. Zhao, B. Mao and M. Cao, *J. Mater. Chem. A*, 2017, **5**, 7001-7014

10 C. Hu, L. Zhang, Z. J. Zhao, A. Li, X. Chang and J. Gong, *Adv. Mater.*, 2018, **30**, 1705538.

11 Y. Zhao, B. Jin, Y. Zheng, H. Jin, Y. Jiao and S.-Z. Qiao, *Adv. Energy Mater.*, 2018, **8**, 1801926.
